# Changes in Metabolic Profile in PLWHIV Switching to Doravirine-Based Regimen

**DOI:** 10.3390/v15051046

**Published:** 2023-04-25

**Authors:** Valentina Iannone, Rosa Anna Passerotto, Francesco Lamanna, Rebecca Jo Steiner, Francesca Lombardi, Pierluigi Francesco Salvo, Alex Dusina, Damiano Farinacci, Alberto Borghetti, Simona Di Giambenedetto, Arturo Ciccullo

**Affiliations:** 1 Dipartimento di Sicurezza e Bioetica, Università Cattolica del Sacro Cuore, Malattie Infettive, 00168 Rome, Italy; 2 Fondazione Policlinico Universitario A. Gemelli IRCCS, UOC Malattie Infettive, 00168 Rome, Italy; 3 Ospedale Belcolle, Medicina Protetta, Unità di Malattie Infettive, 01100 Viterbo, Italy; 4 Ospedale San Salvatore, Dipartimento di Malattie Infettive, 67100 L’Aquila, Italy

**Keywords:** doravirine, NNRTI, metabolic syndrome, dyslipidemia, low density lipoprotein, cardiovascular risk

## Abstract

Thanks to the modern ARV regimens and the fact that the morbidity and mortality of metabolic syndrome increases with age, clinicians are continuously researching effective and safe antiretroviral regimens with low impact on the lipid profile. Doravirine (DOR) is the latest non-nucleoside reverse-transcriptase inhibitor (NNRTI) that shows long-term safety and tolerability and a favorable lipid profile. The aim of this study is to assess the impact of DOR-based three-drug regimens on the lipid profile in clinical practice. We retrospectively analyzed a cohort of 38 treatment-experienced, virologically suppressed people living with HIV (PLWH) switching to this regimen, following the eligibility criteria. We carried out comparison analysis of immunological and metabolic parameters between baseline and 48 weeks of follow up. In our cohort of treatment-experienced, virologically suppressed PLWH, three-drug regimens with DOR showed good efficacy and a positive profile on lipid metabolism at 48 weeks of follow up.

## 1. Introduction

Non-communicable diseases are the real emerging issues among aging people living with HIV (PLWH) on antiretroviral treatment [1]; among them, metabolic syndrome (MS) has become the most relevant, with an estimated global prevalence among PLWH ranging between 16.7 and 31.3% [1,2]. The role played by the HIV infection itself on the genesis of MS is not fully understood; the association with certain ARV molecules, such as protease inhibitors (PIs), has also been studied, observing how their use could lead to an increased risk of metabolic syndrome [3]. Given the increased life expectancy thanks to modern ARV regimens and the fact that the morbidity and mortality of metabolic syndrome increases with age, clinicians are in continuous search of effective and safe ARV regimens with low impact on the lipid profile in order to minimize cardiovascular morbidity and mortality. Although some PLWH are already at high risk of cardiovascular events or diabetes, it is mandatory for healthcare providers to not exacerbate these conditions with antiretroviral therapy administration.

Doravirine (DOR) is the latest non-nucleoside reverse-transcriptase inhibitor (NNRTI) to be introduced for the treatment of HIV-1 infection and has been recommended as part of three-drug regimens by international guidelines for the treatment of HIV-1 infection [4]. DOR is s a pyridinone NNRTI with potent antiviral activity against wild-type HIV-1 and common NNRTI variants, with a favorable pharmacokinetic profile for once-daily dosing, and low potential drug–drug interaction.

Doravirine has an in vitro resistance profile that is distinct from other NNRTI molecules and retains activity against viruses containing the most frequently transmitted NNRTI mutations [5,6]. These data have also been reported from real-life clinical practice, supporting doravirine use in naive patients [4,5,6].

Results from a randomized, active-controlled, double-blind, noninferiority phase 3 trial DRIVE-AHEAD continue to support DOR/3TC/TDF efficacy and its favorable lipid profile at 96 weeks of follow up, compared to EFV/FTC/TDF, as demonstrated at week 48 [7]. In a multicenter, noninferiority trial, DRIVE-FORWARD, results showed the noninferior efficacy of doravirine, compared to ritonavir-boosted darunavir (DRV/r), showing a superior lipid profile at 96 weeks of follow up [8]. Additionally, long-term results from the DRIVE-SHIFT trial [9] have shown a reduction in fasting lipids at 24 weeks after the switch to a DOR-based regimen, and that this reduction is maintained through week 144. The mean weight change from switch to week 144 was +1.4 kg for ISG and +1.2 kg for DSG [9]. According to the aforementioned data and results from the literature, doravirine-based ARV regimens may lead to reduced long-term effects and cardiovascular risk in PLWHIV; however, real-life data from clinical practice are still scarce.

The aim of this study is to assess the impact of DOR-based regimens on the lipid profile in clinical practice during a follow-up time of 48 weeks.

## 2. Materials and Methods

We retrospectively analyzed a cohort of 38 treatment-experienced, virologically suppressed PLWH who switched to a DOR-based 3-drug regimen. We collected clinical history and viroimmunologic and metabolic parameters such as HIV-RNA (copies/mL), T-CD4+ lymphocyte cell count, low-density lipoprotein (LDL) value, total cholesterol, and triglycerides, both at baseline and at 48 weeks of follow up. We applied the following eligibility criteria: patient’s informed consent to data collection, adult age (≥18 years-old), and being on stable ARV with viral suppression (HIV-RNA < 50 copies/mL) at the moment of switch (baseline). We carried out comparative analysis of immunological and metabolic parameters between baseline and 48 weeks via parametric and nonparametric tests and searched for predictors by linear regression. We also analyzed the time to virological failure (VF, defined as a single HIV-RNA determination > 1000 copies/mL or two consecutive determinations > 50 copies/mL) or interruption of the regime (TD, defined as the discontinuation of any drug in the prescribed regimen) via Kaplan–Meier survival analysis, using the Cox Regression Analysis to search for predictors.

## 3. Results

We analyzed a cohort of 38 PLWH: 23 (60.5%) were males, with a median age of 53 years (IQR 46–59) and a median time from HIV diagnosis of 14.3 years (IQR 5.8–21.8). The median time of ARV exposure was 12.2 years (IQR 5.5–18.8) and median time of virological suppression was 8.4 years (IQR 3.9–16.1). All PLWH started an XTC/TDF + DOR regimen, except one of them who started DOR plus FTC/TAF. The major reason for switching to a doravirine-based regimen in our population was the need for a simplified strategy (n 25, 65.8%). Full population characteristics are shown in Table 1.

During a follow-up time of 42.79 patient-years of follow up (PYFU), we observed one VF, a rate of 2.3 per 100 PYFU. The probability of maintaining virological suppression at 48 weeks was 96.9%. We did not find any predictor of VF in our regression analyses.

Regarding treatment discontinuations, we recorded three TD during 43.24 PYFU: one due to hypersensitivity reaction and two due to simplification to a 2DR. The estimated probability of maintaining the DOR-based regimen was 91.7% at 48 weeks. We did not find any predictor of TD in our regression analyses.

Regarding immunological parameters, we observed a reduction in absolute CD4+ count at 48 weeks (median −37 cell/mm^3^, *p* = 0.011), not associated with significant changes in the CD4/CD8 Ratio. Regarding metabolic parameters, LDL, HDL, and triglycerides median values at baseline are shown in Table 1. At 48 weeks of follow up, the median values of the metabolic parameters were: LDL 105.00 mg/dL (IQR 81.00–133.00); HDL 47.9 mg/dL (IQR 44.07–70.4); and triglycerides 94.42 mg/dL (IQR 61.73–133.38). We registered a significant reduction in total cholesterol values at 48 weeks (median reduction –17 mg/dL, *p* = 0.001) and a trend in the reduction in triglycerides (median −16 mg/dL, *p* = 0.052). The only predictor of the reduction in total cholesterol was baseline total cholesterol (for 10 mg/dL more, B −3.3, 95% CI −5.7 a −0.9, *p* = 0.009).

## 4. Discussion

In our cohort of treatment-experienced, virologically suppressed PLWH, three-drug regimens with DOR showed good efficacy and a low rate of discontinuation. Our findings are in line with results from clinical trials [6,7,8,9], which show that DOR-based regimens, compared to older NNRTIs, appear to be an improvement in terms of both efficacy and safety. Moreover, a DOR-based regimen also results particularly appealing as a switch strategy, given its optimal tolerability and its low impact on the lipid profile [10,11]. 

Our data also highlight a positive profile on lipid metabolism, resulting in a median reduction of −17 mg/dL of total cholesterol at 48 weeks of follow up. We also found a reduction in absolute CD4+ cell count, with a median reduction of −37 cell/mm^3^ (*p* = 0.011) not associated with significant changes in the CD4/CD8 ratio. Based on the small number of patients enrolled, the short follow-up time, and the non-change in the CD4/CD8 ratio, the resulting median reduction in CD4+ cell count should not be a concern. We aim to continue the follow up until 144 weeks post-treatment to clarify whether this reduction in CD4+ cell count is confirmed.

Metabolic syndrome is especially concerning in PLWH, even those on stable ARV treatment, given the multiple overlapping risk factors with the seronegative population. HIV infection itself represents a cardiovascular risk factor, due to the HIV-associated low-grade-inflammation and immune dysregulation [12,13,14,15]. Subsequent treatment with antiretroviral therapy is often associated with lipid profile perturbations and increasing levels of pro-inflammatory lipid species, such as oxidized low-density lipoprotein (LDL) and decreasing high-density lipoprotein (HDL) [16]. An overview and management of this bidirectional system in which inflammation increases lipid levels and promotes their modification, sustaining inflammation instead, is needed. It can contribute to understanding pathogenesis and preventing cardiovascular events in this population [17]. 

Lifestyle interventions on PLWH are the first steps needed, leading to reducing the common risk factors such as smoking, drug abuse, and physical inactivity; this can also lead to a reduction in the combination of pharmacotherapies with lipid-lowering medications including statins and fibrates for dyslipidemia, and mitigate CVD risk. The European Society of Cardiology guidelines recommend lipid-lowering therapy to reduce low-density lipoprotein cholesterol to <70 mg/dL in PLWH [18].

In PLWH with metabolic syndrome, reducing cholesterol and triglycerides values is mandatory in order to obtain a reduction in the inflammation degree and the risk of atherosclerotic plaque formation [12,13].

For all these mechanisms mentioned above, using ARV regimens with low impact on metabolic parameters and on the lipid profile is crucial in HIV infection management, and could also lead to finding the optimal combination strategy to improve the quality of life and life expectancy of PLWH [19].

Given the current issues and the aging PLWH population, our results can help in developing tailored therapy, choosing the adequate ARV regimen, and improving clinicians’ ability to deliver optimal preventive care.

The main limitations of our study are the small sample size, the limited follow up, and the lack of a control group. However, real-life data are particularly needed, given their impact on the day-to-day activity of clinical healthcare providers.

In conclusion, our data show the favorable metabolic profile of a two NRTI + DOR regimen in PLWH, leading to a significant reduction in total cholesterol in as soon as 48 weeks. More studies are needed, adding to the current literature, to quantify the benefits of switching to a DOR-based regimen in terms of reducing cardiovascular risk, and reducing the number of cardiovascular events in PLWH.

## 5. Conclusions

In conclusion, our data show the favorable metabolic profile of a two NRTI + DOR regimen in PLWHIV, leading to a significant reduction in total cholesterol in as soon as 48 weeks. More studies are needed, adding to the current literature, to quantify the benefits of switching to a DOR-based regimen in terms of reducing cardiovascular risk, and reducing the number of cardiovascular events in PLWHIV.

## Figures and Tables

**Table 1:** Baseline characteristics of patients.

Variables	n = 38
Sex male, n (%)	23 (60.5)
Age, median (IQR)	53 (46–59)
Risk factor, n (%):	
- MSM *	18 (47.4)
- Heterosexual	13 (34.2)
- PWID *	6 (15.8)
- Other/Unknown	1 (2.6)
CDC, C, n (%)	14 (36.8)
HCV, n (%)	4 (10.5)
HBV, n (%)	4 (10.5)
Zenith HIV-RNA Log_10_/cp/mL, median (IQR)	5.33 (4.70–5.70)
Nadir CD4+ cell/mm^3^, median (IQR)	102 (24–231)
Years of HIV infection, median (IQR)	14.3 (5.8–21.8)
Years of ARV *, median (IQR)	12.2 (5.5–18.8)
Years of virological suppression, median (IQR)	8.4 (3.9–16.1)
LDL cholesterol at baseline, median (IQR)	122 (IQR 106.3–138.8)
HDL cholesterol at baseline, median (IQR)	52.31 (IQR 43.04–63.09)
Triglycerides at baseline, median (IQR)	119.09 (IQR 81.77–155.93)
Individuals on hypolipemic therapy, n (%)	2 (5.3)
Reasons of switching to DOR, n (%):	
- Simplification	25 (65.8)
- Dyslipidemia	1 (2.6)
- Toxicity	10 (26.3)
- Other/Unknown	2 (5.3)

* MSM, Men who have sex with men; PWID, people who inject drugs; ARV, antiretroviral therapy.

## Data Availability

All the data used in this study will be made available upon request.
